# Nanoemulsion Formulations of Fungicide Tebuconazole for Agricultural Applications

**DOI:** 10.3390/molecules21101271

**Published:** 2016-09-26

**Authors:** Vianney Díaz-Blancas, Dora I. Medina, Erika Padilla-Ortega, Raquel Bortolini-Zavala, Melissa Olvera-Romero, Gabriel Luna-Bárcenas

**Affiliations:** 1 Cinvestav Queretaro, Libramiento Norponiente 2000, Fracc. Real de Juriquilla, Queretaro, Queretaro 76230, Mexico; vian_versus@hotmail.com (V.D.-B.); rachel_p53@hotmail.com (R.B.-Z.); melissa_ireth7pr@hotmail.com (M.O.-R.); 2 Tecnologico de Monterrey, School of Engineering and Sciences, Atizapan de Zaragoza, Estado de Mexico 52926, Mexico; dora.medina@cantab.net

**Keywords:** tebuconazole, nanoemulsion, organic-to-surfactant ratio

## Abstract

Tebuconazole (TBZ) nanoemulsions (NEs) were formulated using a low energy method. TBZ composition directly affected the drop size and surface tension of the NE. Water fraction and the organic-to-surfactant-ratio (R_O/S_) were evaluated in the range of 1–90 and 1–10 wt %, respectively. The study was carried out with an organic phase (OP) consisting of an acetone/glycerol mixture containing TBZ at a concentration of 5.4 wt % and Tween 80 (TW80) as a nonionic and Agnique BL1754 (AG54) as a mixture of nonionic and anionic surfactants. The process involved a large dilution of a bicontinuous microemulsion (ME) into an aqueous phase (AP). Pseudo-ternary phase diagrams of the OP//TW80//AP and OP//AG54//AP systems at T = 25 °C were determined to map ME regions; these were in the range of 0.49–0.90, 0.01–0.23, and 0.07–0.49 of OP, AP, and surfactant, respectively. Optical microscope images helped confirm ME formation and system viscosity was measured in the range of 25–147 cP. NEs with drop sizes about 9 nm and 250 nm were achieved with TW80 and AG54, respectively. An innovative low-energy method was used to develop nanopesticide TBZ formulations based on nanoemulsion (NE) technology. The surface tension of the studied systems can be lowered 50% more than that of pure water. This study’s proposed low-energy NE formulations may prove useful in sustainable agriculture.

## 1. Introduction

The increased demand for crop raw materials needed to develop new applications, including biofuels, is creating an increasingly competitive agriculture sector, according to a June 2013 report on worldwide agriculture by the United Nations (UN) Food and Agriculture Organization and the Organization for Economic Cooperation and Development [1]. Consequently, development of more-efficient technologies for formulating pesticides is being sought worldwide [2,3]. Nanotechnology from the food and pharmacy fields are being used to develop a more efficient agrochemical industry featuring nanopesticides that are less harmful to the environment than conventional formulation [4]. However, there is controversy about the fate of nanopesticides and nanofertilizers, including a lack of consensus about a definition of ‘nanopesticide’ that satisfies international regulations.

A nanopesticide may be defined as a nanomaterial containing an active ingredient with pesticidal action [5]. In this regard, nanoemulsions (NEs) [6,7] are of special interest for use as pesticide formulations, since many of them are marketed as emulsifiable concentrates or microemulsions (MEs) [8,9,10] that contain their active ingredient at high concentrations.

NEs are dispersions of two liquid phases whose drop sizes are less than 0.5 microns; the drop size of the discrete phase guarantees mechanical stability [11,12,13]. The advantages of NEs over typical emulsions are their smaller drop sizes, which give the system greater wettability, spreadability, and superior mechanical stability. These characteristics may help reduce volatilization and hydrolysis of the active ingredient, thus augmenting bioavailability. These new properties allow NE formulation with lower concentrations of active ingredients and surfactants [6,14,15,16,17]. NEs generally are obtained by diluting an ME; this process is called phase inversion composition (PIC) and is promoted by the drastic change in the system’s concentration. The emulsification method is designated a PIC when there is inversion of the spontaneous curvature of the surfactant film during emulsification. This low-energy, non-spontaneous NE formulation requires mechanical or chemical energy to disperse the discrete phase as nanodroplets [18].

In addition to active ingredients, formulating pesticides involves solvents and adjuvants whose toxic characteristics may result in formulations more toxic than the active ingredient itself. The advantage of biodegradable solvents is that the toxicity of the final product would not be increased.

**Figure 1:**
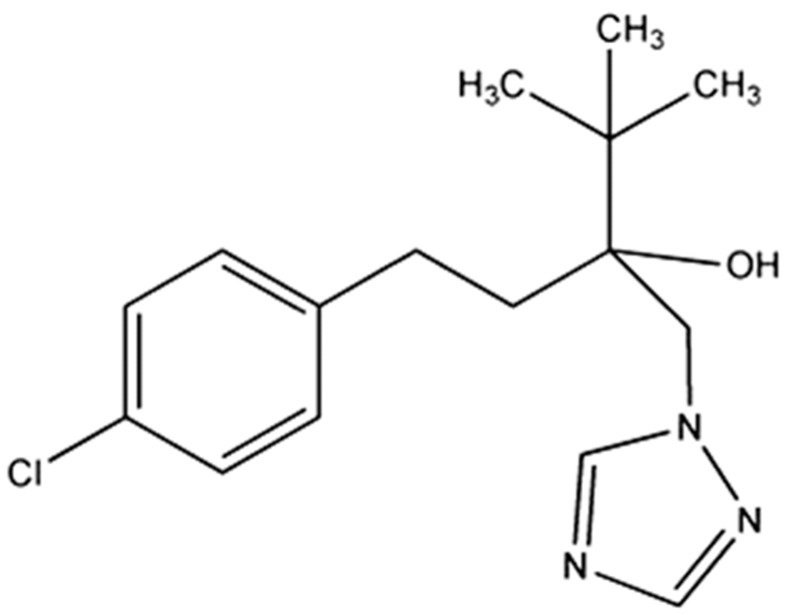
Chemical structure of TBZ.

Commonly used pesticides, including tebuconazole (TBZ), as seen in Figure 1, have this degree of toxicity. TBZ is a fungicide that is on the list of the most dangerous pesticides (Pesticide Action Network International, 2009).

TBZ belongs to the chemical group of triazoles and is noted for its preventive and curative actions. Its disadvantage is low water solubility (32 mg/L); therefore, existing formulations are usually emulsifiable concentrates based on methylene chloride and acetone [19,20].

TBZ has a Groundwater Ubiquity Score (GUS) index of 2.3, which means it is considered as having moderate potential for leaching into groundwater. TBZ is a persistent pesticide with an adsorption constant K_oc_ (for organic carbon) of between 803 and 1251 mL/g [21]. TBZ’s systemic action allows it to be absorbed through plant leaves and roots to inhibit the synthesis of ergosterol, a lipid component of the cell membrane of fungi and micro-organisms. This inhibition prevents the formation of pathogens on the cell walls and slows the growth of germ tubes [20]. There have been other studies about interesting formulations for pesticides [2,22,23,24,25].

The objective of this work was to develop TBZ NEs by constructing a pseudo-ternary phase diagram and identifying TBZ NE concentration regions. This study proposes a low-energy TBZ NE method that may help decrease the required dose of the fungicide and thus the residual active ingredient. The solvents used were less toxic than those currently used, which may help minimize the environmental impact.

## 2. Results and Discussion

### 2.1. Pseudo-Ternary Phase Diagrams

**Figure 2:**
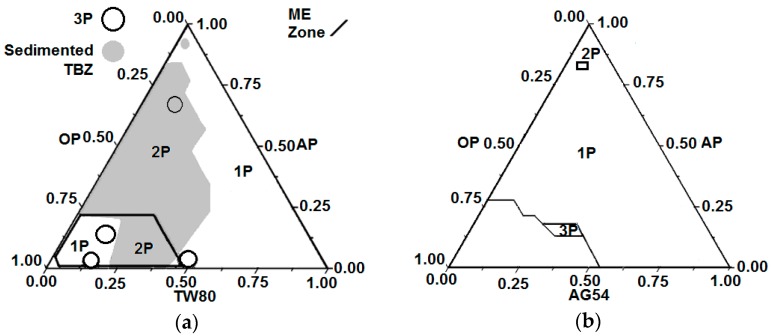
Pseudo-ternary phase diagrams of the systems at T = 25 °C: (**a**) OP//TW80//AP and (**b**) OP//AG54//AP.

An initial requirement for the PIC method to produce NEs is to obtain a bicontinuous ME whose dilution will form an NE [26]. To find the appropriate compositions on the ternary system (OP//SURFACTANT//AP) for phase equilibrium to be achieved and therefore, for an ME to exist, it is necessary to generate a phase diagram. The phase diagrams from the systems studied (OP//TW80//AP and OP//AG54//AP at T = 25 °C) are shown in Figure 2a,b, respectively. Figure 2a shows that over a wide range of compositions, two phases were detected: one solid and one homogeneous liquid. The solid phase was sediment TBZ, and the liquid phase was a liquid equilibrium phase. The sedimentation effect may be due to the higher solubility parameter for the acetone-aqueous phase (AP) pair compared with the acetone-TBZ pair. The ME phase equilibrium area predominated, with composition ranges of 0.49–0.9 from the organic phase (OP), 0.01–0.23 from the AP, and 0.07–0.49 from TW80.

Figure 2b represents the system corresponding to OP//AG54//AP at T = 25 °C. The figure shows the predominance of two liquid phases (2P) in the W/O section. No TBZ sedimentation was observed. As in the previous system, the phase diagram was related to the ratios of the components. In this case, the electrostatic forces were unable to maintain equilibrium between phases. All this caused the presence of areas in which the three liquid phases (3P) were denoted. The ME area corresponded to the compositions of the OP to AP: 0.49–0.90 and 0.01–0.23 and AG54: 0.07–0.49; the O/W section was selected. Although two phases occurred, the proportions were considered in order to compare the methods of NE synthesis by various surfactants.

The difference between the ME areas in the systems studied was due mainly to the nature of the surfactant. In both systems, the interfacial equilibrium was a consequence of the component proportions and the intermolecular forces. The TW80 is a nonionic surfactant that mainly favors Van der Waals forces and hydrogen bonds. The AG54 surfactant is a mixture of nonionic and anionic amphiphilic components, wherein hydrogen bonds occur along with the interactions of attraction and repulsion. As a result of the existing ionic forces, the AG54 system did not present the phenomenon of TBZ sedimentation. However, the presence of electrostatic interactions required more energy input to maintain the balance between phases and avoid their separation as a result of the repulsion among molecules. The stability of the final products (NE) was determined using the polydispersity index (PDI) as the parameter. This parameter determines the particles’ size variability through the dimensionless media from the amplitude of size distribution into the Gaussian bell; it is a function of solution properties, system thermodynamics, and preparation methods. Higher values are indicative of the heterogeneity of the particles’ diameters, and if this increases over time, it could be a consequence of the aggregates’ formation [27]. The PDI can be obtained from dynamic light-scattering measurements (DLS); however, when this parameter is very high the sample is very polydisperse and may not be suitable for DLS measurements. Moreover, the sample contains large particles, aggregates, and dust, which is an indication of an unstable sample through time. The PDI value selected was 0.7; if the PDI of the sample was larger than this value, the sample was discarded.

### 2.2. Microemulsions and Nanoemulsions

Once the composition range of the ME area was identified, the samples were prepared as shown in Table 1. To confirm the formation of the bicontinuous ME, the samples were characterized by optical microscopy to identify whether the system was isotropic or anisotropic. Anisotropic samples are denoted by the birefringence of the sample (liquid crystal conformation) or by differences in the refractive index of the system [28].

**Figure 3:**
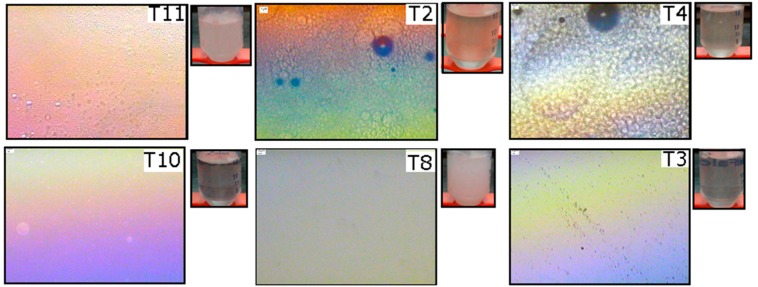
Optical microscope images, with crossed polarizers at 5× magnification, of the ME corresponding to the OP//TW80//AP system at T = 25 °C.

Formulations from various samples were selected by the parameter of translucence, since this indicates the apparent formation of an ME. Figure 3 shows images corresponding to the OP//TW80//AP system at 5× magnification. The table shows that samples T3, T8, T10, and T11 exhibited homogeneity in the refraction of their polarized light, that is, no disparity in the modes by which they refracted light. In addition, samples T2 and T4 showed a shift in brightness, that is, a contrasting color within the same area. However, T4 also showed birefringence, which is characteristic of the formation of a liquid crystal. These results indicate that samples T2 and T4 were anisotropic and that samples T3, T8, T10, and T11 had one refractive index and therefore an isotropic character. This suggests the formation of the ME. The samples were stored for one month to evaluate stability.

**Figure 4:**
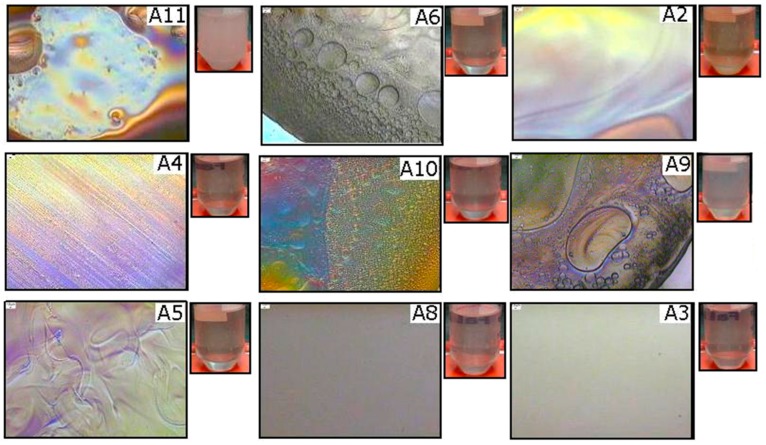
Optical microscope images, with crossed polarizers at 5× magnification, of the ME corresponding to the OP//AG54//AP system at T = 25 °C.

After one month, samples T2, T3, T4, and T10 remained translucent, indicating good stability, whereas samples T11 and T8 showed a white aspect, typical of the formation of macroscopic emulsion. Figure 4 shows images obtained from selected samples from the OP//AG54//AP system and illustrates that samples A2, A4, A5, A6, A9, A10, and A11 showed anisotropy. The presence of the birefringence phenomenon denoted formation of liquid crystals. Furthermore, samples A3 and A8 showed isotropy. Another selected sample of this system was observed to have translucent formulations after one month of storage, meaning that it could be assumed to correspond to an ME. Sample A11 was the exception, as it showed macroscopic emulsions (a white solution).

**Figure 5:**
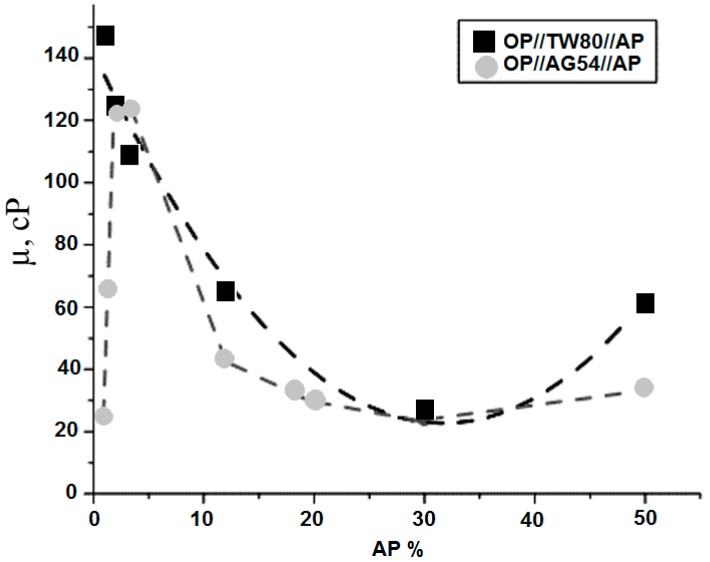
Viscosity of ME as a function of AP % in the OP//TW80//AP and OP//AG54//AP systems at T = 25 °C.

To evaluate the behavior of the systems based on the percentage of the AP, their viscosity was determined. Figure 5 plots the curves for each sample in both systems.

The figure illustrates that at concentrations ranging from 4 to 50 wt %, both studied systems have a similar tendency. When the AP concentration increased from 4 to 30 wt %, the viscosity decreased approximately four-fold. Moreover, when the AP concentration increased from 30 to 50 wt %, the viscosity was augmented, and it can be noted that the system with TW80 enhanced the viscosity more than the AG54 system. In contrast, concentrations ranging from 1 to 4 wt % in samples with AG54 surfactant increased the viscosity up to five-fold. Thus, the minimum viscosity of 27.6 cP (TW80) and 24.3 cP (AG54) corresponded to 30 wt % of the AP in both systems, and a maximum viscosity of 147 cP and 123 cP was obtained with 1 wt % and 2 wt % of the AP of TW80 and AG54, respectively. The minimum viscosity of both systems was for isotropic formulations (T8 and A8), and this minimum confirmed the existence of a transition zone (between 1 and 2 phases, as shown in the pseudo-ternary phase diagrams).

**Figure 6:**
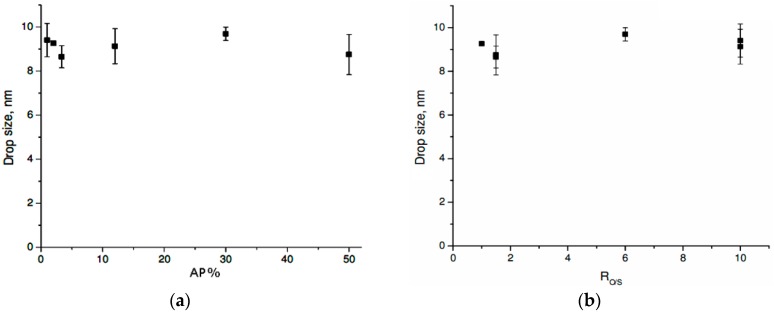
Drop size for the OP//TW80//AP system at T = 25 °C as a function of (**a**) AP % and (**b**) R_O/S_.

The results of the characterization of the bicontinuous MEs from the two studied systems were used to select the composition of the NEs developed. Figure 6a shows the drop size as a function of the AP % for the system OP//TW80//AP. When the AP % was increased from 1 to 50 wt %, the drop size remained 9 ± 1 nm; the system showed no dependence on the concentration of the AP. However, the influence of the organic-to-surfactant-ratio (R_O/S_) parameter could not be ruled out, but Figure 6b shows that the drop size did not vary significantly when the R_O/S_ ranged between 1 and 10.

Drop size primarily was related to the structure of the generated micelles and therefore the type of interactions between these components. The critical micelle concentration (CMC) of the TW80 system was obtained experimentally as 0.094 mmol/L, and the formulations developed in this study had greater surfactant concentrations than the CMC (minimum concentration = 0.3816 mmol/L). Thus, the previously described results confirmed the spontaneous formation of micelles in the system, ideally spherical, as these generally are achieved at higher concentrations than the CMC of the surfactant. Furthermore, the values found for drop size were similar to those of the TW80 micelles without TBZ in the same conditions (10 nm) (results not shown).

**Figure 7:**
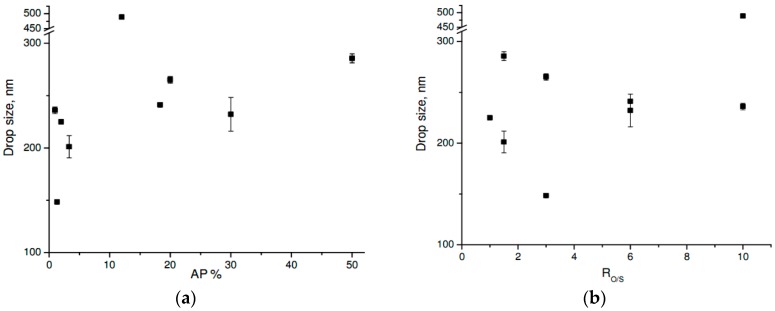
Drop size for the OP//AG54//AP system at T = 25 °C as a function of: (**a**) AP % and (**b**) R_O/S_.

Figure 7a shows the OP//AG54//AP system at T = 25 °C and their drop size as a function of the water concentration in the ME. The strong influence of the AP was observed at percentages below 20 wt %. An increase in the AP from 1.3 to 3.3 wt % increased the drop size from 150 to 200 nm. However, an increase of from 3.3 to 12 wt % more than doubled drop size (from 200 to 488 nm). In addition, greater AP values (from 20 to 50 wt %) did not have a significant effect, with the drop size remaining at approximately 250 nm. Furthermore, as Figure 7b shows, the same R_O/S_ system values resulted in different drop sizes. For example, when the R_O/S_ was 1.5, drop sizes were 285 nm and 201 nm at 50 wt % and 3.33 wt % AP, respectively, and when the R_O/S_ was 10, drop sizes were 236 nm and 488 nm at 1 wt % and 12 wt % AP, respectively. This implies that increased R_O/S_ and AP % in the system affect the overall growth of drop size. Moreover, when the R_O/S_ value was larger, increased AP % affected drop size more, since it was related to the micellar aggregates formed in the NE and their interaction in the system. Specific systems were developed with AG54 concentration values of more than 500 mg/L of surfactant, exceeding the CMC obtained in the laboratory for AG54 (165.9 mg/L). Therefore, spontaneous formation of micelles was considered to exist in the NE.

The NE’s drop size is a function of the quantity and nature of the components, the type of surfactant (nature and molecular weight) and the obtaining method; however, there are also phenomena that promote destabilization [29,30,31].

According to the displayed results, the system with TW80 presents drops sizes much lower than the AG54. This result can be attributed to the fact that TW80 is mainly a hydrophilic nonionic surfactant (HLB = 15); therefore, the types of interactions that may occur with it involve mainly the London forces and hydrogen bonds that occur between polar and nonpolar regions of the chemical groups present in the components, and mainly in the organic phase. In contrast, AG54 is a mixture of nonionic and anionic surfactants, so, in addition to contributing to interactions similar to those in TW80, it contributes strongly to electrostatic forces. The results allow the inference that the presence of electrostatic charges affects the OP/AP interface, destabilizing the hydration layer around the particles and increasing the strength of colloidal repulsion and coalescence. Additionally, this may favor the Ostwald ripening phenomenon, thus generating the increases in drop size [32]. Even more, when the micellar structure of AG54 formed, repulsive forces among the micellar structures that had already formed.

**Figure 8:**
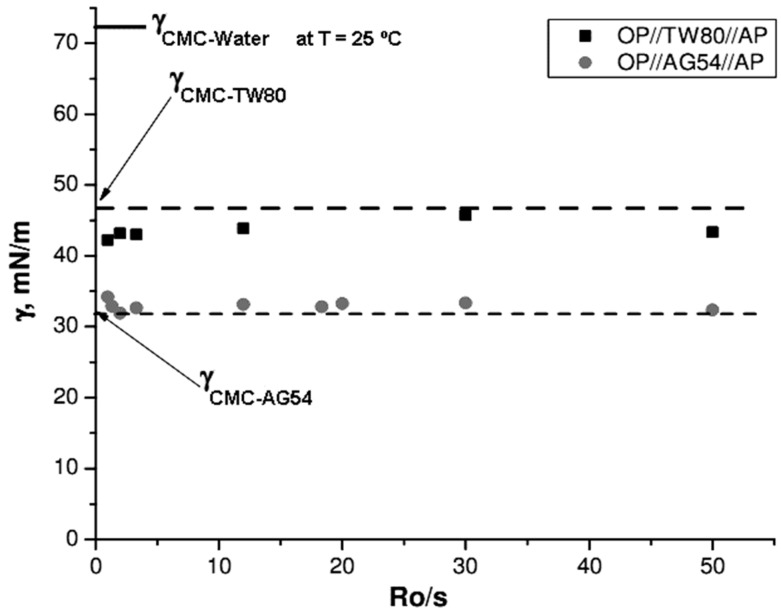
Surface tension of the NE as a function of AP % for the OP//TW80//AP and OP//AG54//AP systems at T = 25 °C.

In addition to drop size, another important feature of the NEs was whether their surface tension decreased compared to the surface tension of the water, which would indicate good wettability of the system in future potential applications. Figure 8 shows results obtained with the AG54 and TW80 systems. The semi-continuous line represents the CMC values obtained for TW80 and AG54.

The TBZ composition directly affected the drop size and surface tension of the NE, both of which were related to the wettability and availability of the active ingredient.

All samples from the TW80 system exhibited very similar surface-tension values from 42 to 45 mN/m, regardless of the R_O/S_. In addition, these values were up to 50% lower than the surface tension of water under the conditions of analysis (γ = 72.8 mN/m) and very similar to the corresponding surface-tension values when the solution had only surfactant in the CMC (γ = 47 mN/m). For example, a minimum surface-tension value might be expected due to the formation of micelles in the sample. This corroborates that, as shown by the drop size, spontaneous micelle formation and stability both existed under these experimental conditions.

The surface tension of the AG54 surfactant system samples showed surface-tension values close to 31 mN/m, without any significant effect from increasing the surfactant concentration and therefore decreasing the R_O/S_. This surface tension value (31 mN/m) was the value of the surface tension at the concentration CMC (CMC = 165.9 mg/L), indicating that this system contained micelle formation in the solution. Unlike the TW80 systems, the AG54 systems had lower surface tensions. Considering surface tension as a manifestation of the cohesive force among a liquid’s molecules, it can be deduced that AG54 formulations have better wettability.

**Figure 9:**
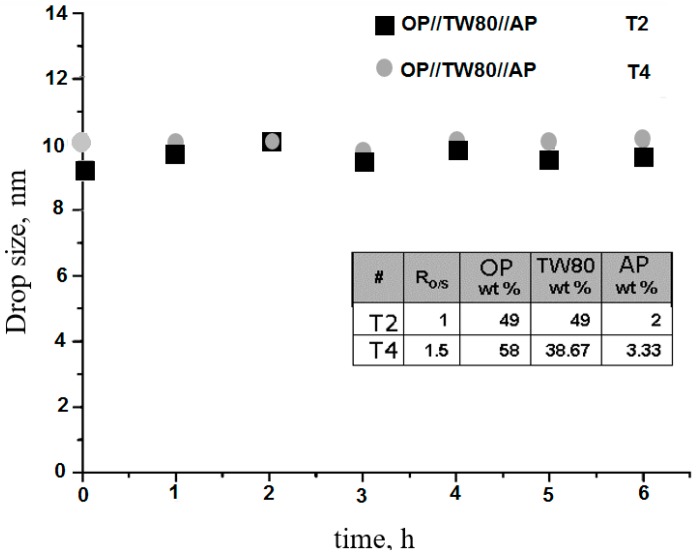
Drop size as a function of time for selected NEs for the OP//TW80//AP system at T = 25 °C.

Finally, the experiments were monitored during the more than six hours required for the development of the corresponding NE. The purpose was to evaluate drop size over time in those formulations characterized by relatively small drop sizes, storage stability (with no phase separation), and non-recrystallization of TBZ. As Figure 9 shows, drop sizes over time for selected formulations of the OP//TW80//AP system at T = 25 °C corresponded to systems that showed anisotropy (T2) and liquid crystal formation (T4).

According to Figure 9, drop size measurements remained unchanged, confirming the stability of NE over the period of time studied.

**Figure 10:**
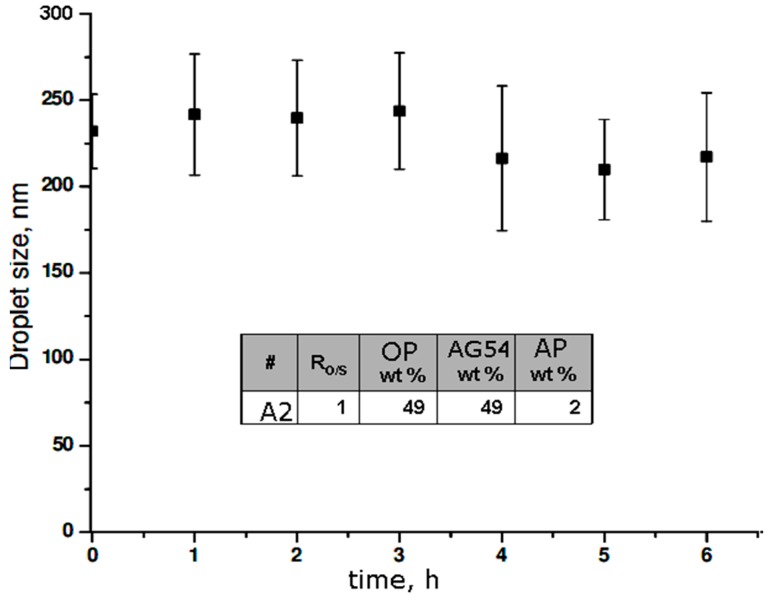
Drop size as a function of time for selected NEs for the OP//AG54//AP system at T = 25 °C.

The selected sample of the OP//AG54//AP system corresponded to the formulation with 2 wt % of the AP and liquid crystal formation in its ME (Figure 10). A drop size of about 225 nm was maintained; however, each attempt had a deviation in measurements of approximately ± 30 nm, which can be attributed to the nature of the surfactant, since it generates interactions, attraction, and repulsion among the polar part and the loads, which may be constantly present in the solution, provoking significant mobility among molecules. Although a similar average drop size was present over a period of six hours, the AG54 showed a considerable instability in formulating the NE, compared with the systems obtained by TW80, whose molecules did not involve net charges. This study found that the selected ME showed the formation of liquid crystals for both systems. The selected ME appears to favor TBZ stability in the system, preventing its sedimentation and thus improving the kinetic equilibrium of the NE.

## 3. Materials and Methods

### 3.1. Chemicals

TBZ fungicide (whose structure is shown in Figure 1), with a purity of 98 wt % and solubility in water of 0.1 g/L and in acetone of >1000 g/L [33,34], was supplied by Velsimex (Mexico City, Mexico). Glycerol and the nonionic surfactant Tween 80 (Polyoxyethylenesorbitan monooleate, MW: 1310 g/mol) [35] were purchased from Sigma Aldrich. Acetone was purchased from JT Baker Company, and commercially available Agnique BL1754—which is a mixture of nonionic and anionic surfactants—was acquired from BASF (a mixture of approximately 30%–60% calcium dodecylbenzenesulfonate and 20%–40% nonylphenol, ethoxylates) [36].

All experiments used deionized water supplied by Meyer Chemical Company. All products were used as received.

These two solvents have a very low degree of toxicity in comparison with the ones used nowadays in industry, such as toluene or xylene [37,38].

### 3.2. Pseudo-Ternary Phase Diagrams

The phase diagrams were determined for the pseudo-ternary system: the OP, AP, and surfactant phase (Tween 80, TW80 or Agnique BL1754, AG54) and constructed through the observed phase behavior and predominant areas. The OP was prepared with 5.4 wt % of TBZ as an active ingredient, 54.9 wt % of glycerol as a co-solvent, and 39.7 wt % of acetone as a solvent; these components were mixed and stirred at room temperature for 24 h before use to ensure homogeneity of the mixture. Initially, experiments were carried out weighing the OP and surfactant (analytical balance APX200; Denver Instrument, Bohemia, NY, USA) in falcon tubes with various Ro/s to map the entire diagram. Then, AP was added and the three-component sample was mixed by stirring with a vortex (G560 GVortex-2 Genie; Scientific Industries, Inc., Bohemia, NY, USA) at T = 25 °C in a water bath (TE-D10; Techne Tempette, Staffordshire, UK) for 24 h. Each point of the diagram was done in triplicate. Areas of ME predominance were recorded for further analysis. Criteria for assigning ME regions were based on the observance of the cloud point (the incipient point of phase separation), that is, the visual transition from a transparent, homogeneous mixture to an opaque or translucent one. To confirm the number of phases, all samples were observed using optical microscopy.

### 3.3. Nanoemulsion Preparation

NE formulations were developed by the PIC method, as previously reported by Wang et al. [23]. Using the information from the pseudo-ternary diagrams, compositions of ME were mapped as shown in Table 1. First, the OP and surfactant were mixed with magnetic stirring, 400 rpm, at T = 40 °C for 30 min (stirring and hot plate, C-MAG HS4; IKA, Wilmington, NC, USA). Then the AP was added at a flow rate of 3 mL/min, heating was turned off, and stirring continued for 40 min. In the final step, samples were stored in closed containers at T = 25 °C. This ME precursor was diluted with water at a ratio of 1:100 and stirred for 1 min on a vortex mixer at room temperature.

### 3.4. Characterization of MEs and NEs

MEs were characterized by determining their viscosity with an Ostwald type viscometer (model NDJ-8S, Changzhou, Jiangsu, China), and images were obtained using an optical microscope (Olympus BX60, Tokyo, Japan) with crossed polarizers (Olympus U-AN360 and U-DICR, Tokyo, Japan) at 5× magnification [39,40]. Further characterization of NEs included drop size and surface tension (γ) at T = 25 °C. Measurements were performed in triplicate. The average drop size was determined using dynamic light-scattering equipment (Zeta Sizer^®^, Malvern, UK). Measurements were performed using the parameters of viscosity in a dispersion medium, water, at T = 25 °C, and the refractive index of the OP = 1.433.

Light-scattering measurements also helped verify that there was no precipitation of TBZ. Surface-tension measurements were determined with a tensiometer (model 703, Sigma, Lichfield, UK) using the corrected Nuoy Ring method.

## 4. Conclusions

This study achieved NE formulation of TBZ as an active ingredient with drop sizes of about 9 nm for the OP//TW80//AP system, with oil-phase concentrations in the precursor ME of up to 90 wt %. Stability values over time showed that with an R_O/S_ of 1 and 1.5, the drop size stayed constant for six hours.

The AG54 systems maintained storage stability with a drop size of between 148 nm and 488 nm, which did not vary with the individual parameters studied. However, the R_O/S_ and AP % together influenced the system. The evolution of drop size over six hours showed instability. Selected MEs formed liquid crystals, according to optical microscope images.

The phase diagrams showed that the balance between phases strongly depended on the nature of the surfactant. The zone O/W was selected to study ME development.

Factors such as the nature of the surfactant’s molecules governed interactions during the formation of micelles, which affected drop size. A larger drop size resulted in less cohesion of the anionic surfactant’s molecules to the solution.

## Figures and Tables

**Table 1:** Composition of different nanoemulsions.

OP//TW80//AP	OP//AG54//AP	R_O/S_^a^	OP (wt %)	S (wt %)	AP (wt %)
T1	A1	1.0	5.0	5.0	90.0
T2	A2	1.0	49.0	49.0	2.0
T3	A3	1.5	30.0	20.0	50.0
T4	A4	1.5	58.0	38.7	3.3
T5	A5	3.0	60.0	20.0	20.0
T6	A6	3.0	74.0	24.7	1.3
T7	A7	6.0	50.0	8.3	41.7
T8	A8	6.0	60.0	10.0	30.0
T9	A9	6.0	70.0	11.7	18.3
T10	A10	10.0	80.0	8.0	12.0
T11	A11	10.0	90.0	9.0	1.0

^a^ R_O/S_: organic-to-surfactant-ratio.
